# A Mixed Methods Evaluation of a Digital Intervention to Improve Sedentary Behaviour Across Multiple Workplace Settings

**DOI:** 10.3390/ijerph17124538

**Published:** 2020-06-24

**Authors:** Bradley MacDonald, Ann-Marie Gibson, Xanne Janssen, Alison Kirk

**Affiliations:** School of Psychological Sciences and Health, University of Strathclyde, 16 Richmond Street, Glasgow G1 1XQ, UK; annmarie.gibson2015@gmail.com (A.-M.G.); xanne.janssen@strath.ac.uk (X.J.); alison.kirk@strath.ac.uk (A.K.)

**Keywords:** RE-AIM, sitting time, office workers, process evaluation, workplace health

## Abstract

Background: Prolonged sedentary behaviour (SB) is associated with risk of chronic diseases. Digital interventions in SB require mixed method evaluations to understand potential for impact in real-world settings. In this study, the RE-AIM QuEST evaluation framework will be used to understand the potential of a digital health promotion application which targets reducing and breaking up SB across multiple workplace settings. Methods: Four companies and 80 employees were recruited to use a digital application. Questionnaires were used to measure SB, and additional health and work-related outcomes at baseline, one month, three month and six month follow-up. Qualitative data was collected through focus groups with employees and interviews with stakeholders. Questionnaire data was analysed using Wilcoxon Sign Rank tests and qualitative data was thematically analysed. Results: The digital application significantly increased standing time at one month for the total group and transitions per hour in one of the companies. Facilitators and barriers were identified across RE-AIM. Conclusions: Addressing the barriers which have been identified, while maintaining the positive attributes will be critical to producing an effective digital application which also has the potential for impact in the real world.

## 1. Introduction

Sedentary behaviour or sitting time is any waking behaviour which is under 1.5 metabolic equivalents done whilst seated or in a recline position [1]. Accumulating high daily amounts of sitting time is associated with increased risk of cardiovascular disease, type 2 diabetes, all-cause mortality, some cancers, and reduced mental wellbeing [2,3,4,5]. Office workers accumulate large amounts of sitting time [6,7], and are therefore at particular risk. 

Digital interventions to reduce sitting time in office workers may have the potential to reach large populations of employees for minimal resources; however, little is known of this potential as most interventions report only on the effect on behaviour; and very few evaluate for wider potential for impact across settings, under real-world conditions [8,9,10]. Researchers have suggested that building collaborations within the health industry is the best way forward, as collaborations would enable the sharing of expertise and resources. This should maximise the capacity to adequately test a technology’s potential to influence behaviour in the workplace [11,12]. Additionally, experts have suggested that these evaluations should be performed in early phases of research [13,14,15], and evaluate multiple indicators of real-world potential, to enable early understanding of the adaptations which may be needed to have large scale, real-world impact [8,13,15,16,17]. This approach, however, would require appropriate evaluation methods, and in two recent reviews of digital workplace sedentary behaviour interventions, both authors advocated for the use of evaluation frameworks that support mixed methods study designs to more robustly plan, conduct and report digital interventions in office workers [8,9]. 

The RE-AIM framework, aided by Qualitative Evaluation for Systematic Translation (QuEST), provides a mixed methods evaluation framework which can support a robust evaluation of the potential for wider impact by examining indicators across five distinct intervention dimensions (R—reach, E—effectiveness/efficacy, A—adoption, I—implementation and M—maintenance) [11,18]. Reach is defined as the absolute number, proportion, and representativeness of eligible individuals who participate in a given initiative. Effectiveness/efficacy assesses the impact of an intervention on the relevant outcomes, including potential adverse effects, quality of life, and economic outcomes. Adoption assesses the reach and effectiveness/efficacy of an intervention at the setting level. It is defined as the absolute number, proportion, and representativeness of the settings and intervention agents (a group of people who implement the intervention) who are willing to initiate a program. Implementation refers to the intervention agents’ fidelity to the various elements of an intervention’s protocol. This includes consistency of delivery as intended; and the time and cost of the intervention. The maintenance dimension is concerned with both setting level indicators, and the individual level indicators. At the setting level, maintenance is the extent to which a program or policy becomes institutionalised or part of organisational practices and policies. At the individual level, maintenance is assessed by monitoring of effectiveness of an intervention or program six months or more after the most recent contact [11,19]. Forman and colleagues added QuEST to RE-AIM to guide qualitative inquiry to further explore the dimensions of RE-AIM [18]. 

In this study, the RE-AIM QuEST evaluation framework will be used to examine and understand the potential reach, effectiveness, adoption, implementation and maintenance of a digital health promotion application which targets reducing and breaking up sedentary behaviour across multiple workplace settings. 

## 2. Materials and Methods

### 2.1. Collaboration

A collaboration with the digital health company, Welbot, was established in the early phases of the development of the Welbot digital application aimed at improving wellbeing in the workplace. The ultimate aim of this collaboration was to create a digital intervention that was evidence-based and uniquely tailored to each individual user. Through this collaboration, the research team provided expertise across two project phases. In the first phase, the research team analysed existing content, and created and analysed new intervention content. Firstly, the research team validated previously developed content including stretches, exercises and mindfulness nudges. A nudge is a notification that asks users to engage in a simple 1–5 min activity aimed at reducing and breaking up their sitting time (e.g., perform an exercise, make a mindful cup of tea). Validation involved providing a quality score, correlating specific nudges with the evidence-base, and suggesting recommendations for improvement. Secondly, the research team created new content for the digital intervention, which resulted in approximately 532 new nudges. The final part of phase one was content analysis. This involved collating all content into 4-week progressive journeys that were either more physically orientated (e.g., ‘Stand Up, Sit Less, and Move More’ or ‘Less Time on Screens’) or mentally orientated (e.g., ‘Reduce Stress’ or ‘Reduce Procrastination’). The second phase of the collaboration focused on testing the digital application, part of which, was to evaluate Welbot in the real-world across multiple settings. This is the focus of this paper. 

### 2.2. Intervention

**Figure 1:**
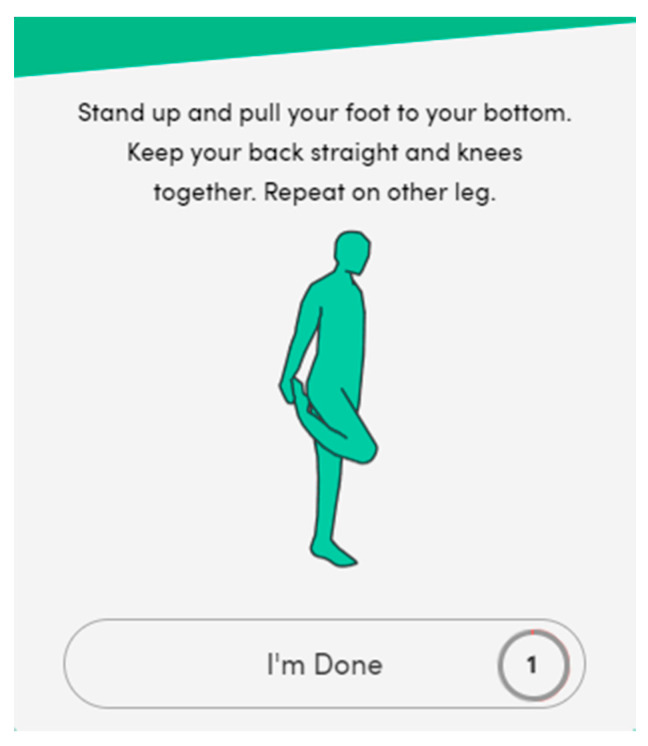
Example of nudge ‘doing card’ delivered during the intervention.

The digital application is downloaded by individual users onto their work computer, and incorporates activities such as stretching, exercises, screen breaks and mindfulness; which are delivered to users in the form of ‘nudges’. Each ‘nudge’ has three phases; a ‘preparation card’ which explains what the nudge will require; a ‘doing card’ which explains and visually demonstrates how to perform the nudge; and a ‘done card’ which explains why the nudge is good for physical and/or mental wellbeing. Figure 1 illustrates an example nudge from the Welbot digital application. 

### 2.3. Study Design

This study utilises both qualitative (interviews and focus groups) and quantitative (questionnaire) methods to collect data across the five RE-AIM dimensions. Data collection was informed and guided by the RE-AIM QuEST mixed methods framework for program evaluation.

### 2.4. Recruitment

Ethical approval was obtained from the university ethics committee and, after this, a contact list of companies was developed by the research team and Welbot. A convenient sample of 18 companies with primarily office-based employees were approached via email to participate in the study. Within each company, a gatekeeper was approached to send a participant email to all eligible employees. Each employee received an email with a participant information sheet, and was asked to attend a brief presentation explaining how to download and use the desktop-based application. After the presentation, employees were given the option to sign informed consent in person or respond via email at a later point. Employees were eligible if they were adults; employed full time or part time at the company and spent the majority of their working day seated using a computer. Employees were excluded if they were not 18 or had a physical health issue (e.g., severe back pain) that would affect their ability to alter their sedentary behaviour. 

### 2.5. Data Collection

Information relevant to indicators of adoption and reach was collected during the recruitment process. For example, in relation to adoption, the lead researcher (B.M.) recorded: the number of companies approached to participate, company size, the recruitment methods used and any reasons given for not participating. The quantitative data were collected at baseline, one month, three months and six months. The qualitative data (participant focus groups and stakeholder interviews) were collected after three months of using Welbot. The primary effectiveness outcomes measured were breaks in sedentary behaviour at work, and total sedentary behaviour at work. Secondary efficacy/effectiveness outcomes included musculoskeletal pain, health related absenteeism and engagement in work. 

#### 2.5.1. Questionnaire Data Collection

After providing informed consent, participants were sent an email with a participant identification number and a baseline questionnaire via Qualtrics. The questionnaire data was collected in the same manner at one month, three month and six month follow-up time points.

##### Questionnaires

The OSPAQ (Occupational Sitting and Physical Activity Questionnaire) was used to measure effectiveness on occupational sitting time. It is a brief instrument which measures the percentage of work time spent sitting, standing, walking, and doing heavy labour, as well as the total length of time (in hours) worked in the past seven days. This questionnaire has been reported to have acceptable reliability and validity for application in office-based studies [20,21]. Additionally, as a measure of breaking up sedentary behaviour participants were asked, via the Qualtrics questionnaire, how many times they stand up from their desk per hour and per day. 

The NMQ (Nordic Musculoskeletal Questionnaire) (Cronbach’s alpha = 0.854) was used to measure pain across nine items (neck, shoulders, elbows, wrists/hands, upper back, lower back, hips/upper leg, knees and ankles). Participants were asked if they experienced pain in the past seven days across the nine items indicating pain = 1 or no symptoms = 0. A pain score was calculated by totalling the participant’s responses across the nine items, with a maximum pain score of nine and a minimum pain score of zero. NMQ has been used to assess pain in a variety of workplace settings, including desk-based employees, and is considered valid and reliable for use as a screening and surveillance tool for musculoskeletal pain [22,23]. 

The validated UWES-17 (The Utrecht Work Engagement Scale Questionnaire) was used to measure changes in engagement in work [24]. It measures three dimensions of engagement: vigour, dedication and absorption [25]. Each of the 17 items (Cronbach’s alpha = 0.905) is scored on a seven-point rating scale from zero (never) to six (always). A mean engagement score is calculated for each participant, with a range from zero (no engagement) to six (always engaged). 

The short form of the validated HPQ (Health and Work Performance Questionnaire) was used to measure health related absenteeism and presenteeism [26,27]. The short form consists of six questions and enables a calculation of net change in absenteeism. 

#### 2.5.2. Qualitative Data Collection

##### Focus Groups with Intervention Participants

All intervention participants were e-mailed to further participate in a focus group. A convenient sample (*n* = 16) agreed to take part and signed a second consent form. A semi-structured topic guide was developed to explore the participants’ perceptions of using the Welbot application in line with the RE-AIM QuEST mixed methods framework. The topic guide was developed, piloted and refined prior to the focus groups and interviews taking place. Focus groups were approximately 45 min in length. 

##### Interviews with Stakeholders

Stakeholders were identified through the recruitment process and were emailed to be a part of the evaluation. Four interviews were conducted in person with at least one stakeholder from each participating company. A stakeholder interview guide was developed in line with the RE-AIM QuEST framework, and adapted for each interview based on the stakeholder’s position in the company, and involvement with aspects of the intervention. The interviews varied in length (15–60 mins).

### 2.6. Measures

Table 1 illustrates each of the dimensions and indicators assessed in this process evaluation, along with the measures used for assessment. Each indicator corresponds to a dimension of RE-AIM and helps to explain that dimension. The measure column indicates the data source used to inform the corresponding indicator of RE-AIM.

### 2.7. Data Analysis

#### 2.7.1. Questionnaire Data Analysis

The data collected from all questionnaires was downloaded from Qualtrics survey program into excel where incomplete or missing data was removed. The cleaned data was then extracted into SPSS statistical analysis tool, and analysed. Visual inspection of the Q-Q plots and box plots revealed outliers in the OSPAQ data set. This was due to participant error when self-reporting the percentage of their working day spent sitting, standing, walking and performing heavy labour. The total percentage of all four categories should equate to 100%. However, certain participants reported totals below or above 100% therefore, totals that were ≥ 90% or ≤ 110% were used as cut of points and values outside these were not included in the analysis of the OSPAQ. Data were checked for normality using skewness and kurtosis measures and Shapiro–Wilk tests [28,29,30]. The skewness and kurtosis z values were checked by dividing the value by its standard error to see if it fell between −1.96 and +1.96. Histograms were also generated and visually inspected for skewness and kurtosis. These analyses showed the majority of the data was not normally distributed, and therefore non-parametric Wilcoxon Sign Rank tests were conducted [28,29,30,31,32]. A Bonferroni correction was made for the three data collection time points, and alpha value was set at 0.0167. A value under 0.05 but over 0.0167 was categorised as trending toward significant.

#### 2.7.2. Qualitative Data Analysis

Braun and Clarke’s thematic analysis (TA) approach [33] was used to separately analyse both the study participant data and the stakeholder data. This approach was selected for its adaptability to different types of interview data. It also enabled the use of deductive coding, based on the RE-AIM framework [33,34]. Firstly, the lead researcher (B.M.) familiarised themselves with the data. As the lead researcher understood their central place in the interpretation of the data [35], this process started by listening back to the recordings after completion of the focus groups and interviews and creating reflexivity notes [35]. The interviews were then listened to again and transcribed verbatim. The transcripts were uploaded onto an analysis software tool Nvivo (12) to facilitate organisation of the coding process. The lead researcher read each of the transcripts and pulled together text that the lead researcher considered analytically important and created initial codes. Deductive coding was carried out in relation to the RE-AIM QuEST framework, aligning data to one of the five indicators of the framework. A second sweep of coding was conducted to enhance trustworthiness [34]. Additionally, to enhance rigour, the lead researcher, along with another experienced qualitative researcher and critical friend (A.M.G.), interrogated the lead researcher’s initial interpretation of the data [36,37]. This coding process was used first for the intervention participant data, and then repeated in a second analysis of the stakeholder data. After the completion of the second coding sweep, similar coding constructs were brought together into initial themes and renamed. The themes were reviewed and after reflecting on the feedback, the lead researcher revisited the theme constructs and subsequently renamed and defined each theme. Quotes were then selected which best illustrated the central organising concept within each theme. 

## 3. Results

Eighty employees (24 males, 55 females and one non binary person) between the ages of 20 and 65 completed the baseline questionnaire. The mean age of participants was 34 years (SD = 11.2 years, (male = 35.3 years, SD = 11.7, female = 33.4 years SD = 11.2)), with mean working hours of 38.3 h per week (SD = 7 h/ week), and mean working days of 4.8 days per week (SD = 0.8 days/week). The sample was predominantly white European (*n* = 76). On average, participants report to be sitting for 77.3% of their working day at baseline. All baseline descriptive statistics are presented in Table 2.

Additionally, 16 of the above-mentioned participants took part in one of three focus groups. Five stakeholders across the four participating companies also took part in interviews. The five stakeholders had various roles within each company, including: human resource manager, company director, managing director, human resources and business officer and director of operations. In the results section, quantitative and qualitative indicators are presented within the dimension of the RE-AIM framework to clearly illustrate where data or information relates to individual dimensions. The dimension order was altered to Adoption, Reach, Implementation, Effectiveness and Maintenance to more accurately reflect the chronological order of occurrence within the research process.

### 3.1. Adoption

Four small/medium sized companies with offices in Edinburgh and Glasgow, United Kingdom agreed to participate in the study. Of the 18 companies that were approached via email, nine responded asking for more information, which was provided via email. Two companies decided not to participate at this stage and three companies did not respond to further emails. Companies that decided not to participate reported IT system changes, existing programs and workload pressures as reasons for not adopting the intervention. Companies that did not adopt were also significantly larger than companies that adopted the intervention and required significantly more email and phone meetings. After the analysis of the focus groups and interviews, one theme developed relating to the adoption dimension. The theme ‘Company buy-in for wellbeing’ is presented in Table 3, along with participant quotes which illustrate the theme. 

### 3.2. Reach

#### 3.2.1. Participation Rate

In total, of the 137 employees across the four companies who were eligible to participate in the study, 80 enrolled and completed the baseline questionnaire. This equalled to approximately 59% of the original eligible employee population. The individual company participation rates are presented in Table 4. In summary, Table 4 shows a high variation in participation rate between the four companies. Companies with higher participation rates were smaller and had more managers participating in the intervention. 

#### 3.2.2. Dropout Rate

Of the 80 participants that completed baseline questionnaires, 60% (*n* = 48) completed one month follow-up, 42% (34) completed three month follow-up and 31% (25) completed six month follow-up. This information along with individual company dropout rates are presented in Table 5. 

#### 3.2.3. Barriers and Facilitators to Reach

One theme developed as a result of the analysis of the qualitative data relating to the reach dimension. The theme ‘Existing awareness that sitting is a health issue to address’ is presented in Table 6, along with participant quotes which illustrate the theme. 

### 3.3. Implementation

#### 3.3.1. Cost

Welbot’s monthly price ranges from £1 to £2.50 per person, depending on the size of the organisation, and length of the contract. Special category customers, such as social enterprises, charities and educational institutions qualify for the lowest price. Welbot did not charge the participating companies for using their program during the intervention; therefore, using the company characteristics, Welbot provided the estimated financial investment for each company over the intervention period. This is presented in Table 7. This estimate is based on 100% retention of the participant population. Additionally, each company self-reported the total hours of company time spent implementing the intervention and this is also presented in Table 7. The table illustrates the estimated financial cost of the intervention, as well as the time used by each company to implement the intervention. Additionally, the average cost and time used, per company, and per participant, is presented. Stakeholder interviews revealed that this time was allocated to the following tasks: IT set up and checks; emails and meetings with the primary researcher, and internal meetings and promotion of the intervention. 

#### 3.3.2. Facilitators and Barriers of Implementation

Four themes developed as a result of the analysis of the qualitative data relating to the implementation dimension of RE-AIM. The results are presented in Table 8. The table shows the four themes along with example quotes from participants and stakeholders which illustrate what participants shared in relation to each theme. 

### 3.4. Effectiveness

#### 3.4.1. Primary and Secondary Outcomes

Company 4 was not included in the individual company analysis because of the high dropout rate. Wilcoxon Sign Rank tests were deemed inappropriate to perform for absenteeism due to the high volume of zero values which were recorded at all time points. Mean values for absenteeism for each time point will be presented to show that there was no change throughout the study. 

##### Sitting, Standing, Walking and Transitions

**Figure 2:**
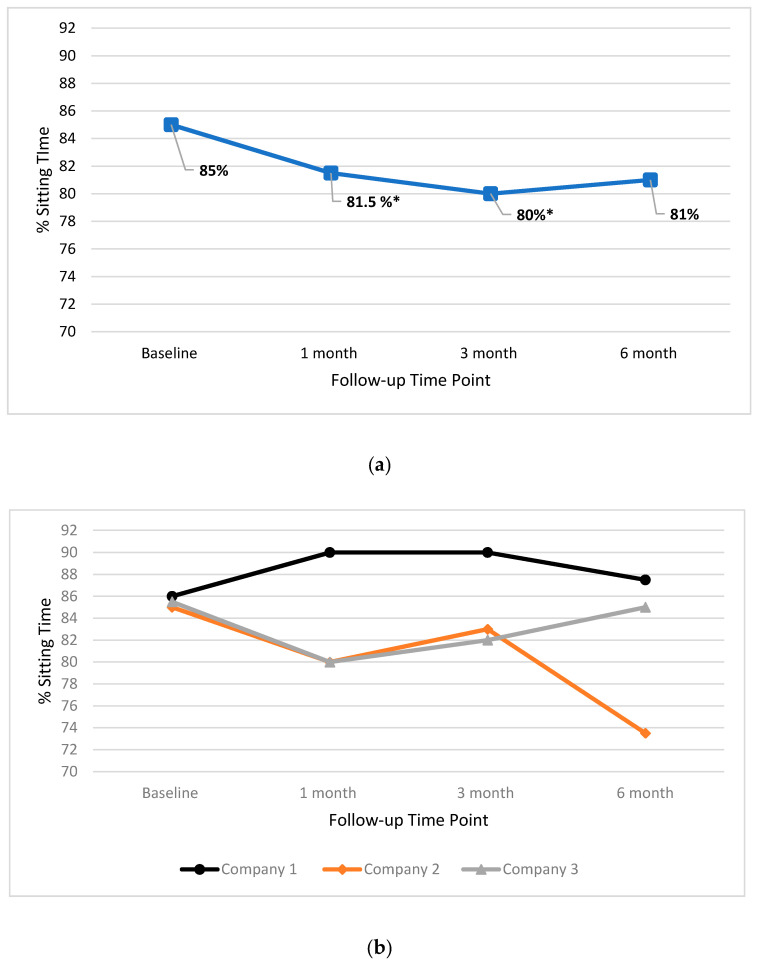
Median percentage workplace sitting time for the total group (**a**) and individual companies (**b**) at baseline, one month, three month and six month time points. * = trend towards significant change.

Figure 2 illustrates median sitting percentage for the total group (Figure 2a), and individual companies (Figure 2b) for all time points. In the total group the baseline median for matched pairs was 85%. Results show that the median sitting time reduced by 3.5% at one month follow-up, and by 5% at three month follow-up. Results of the related samples’ Wilcoxon Sign Rank test indicated a trend towards significant change at both one month (Z = −1.989, *p* = 0.047), and three month (Z = −2.191, *p* = 0.028) compared to baseline. No trends were seen for other time points (Figure 2a) or individual companies (Figure 2b). 

**Figure 3:**
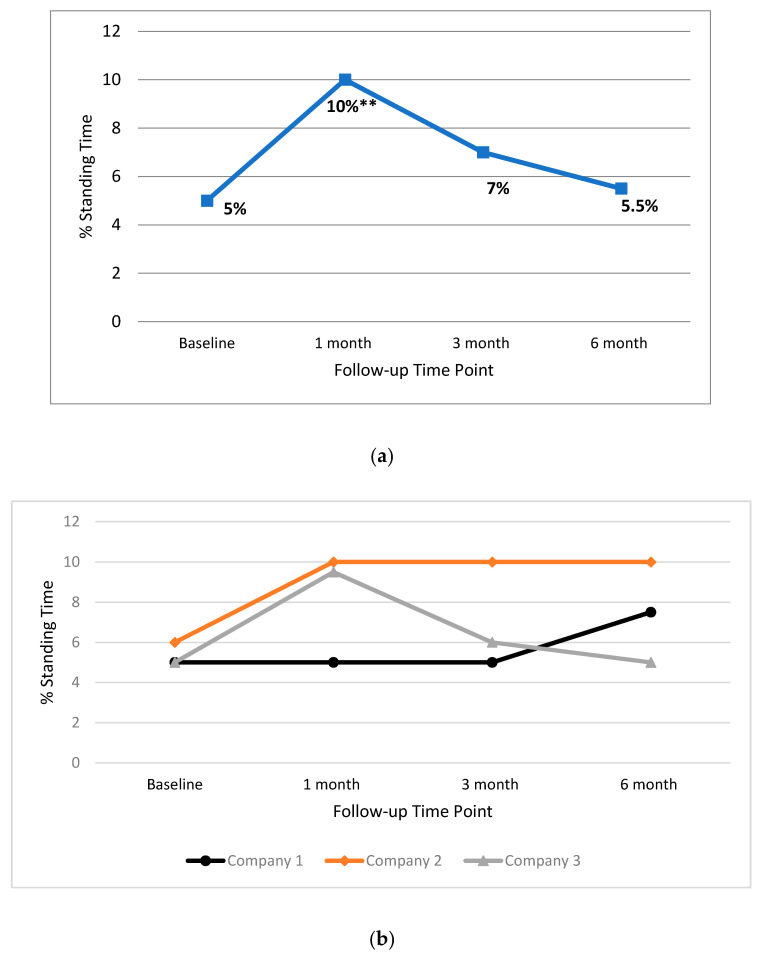
Median percentage workplace standing time for the total group (**a**) and individual companies (**b**) at baseline, one month, three month and six month time points. ** = significant change.

In Figure 3, the median standing percentages for the total group (Figure 3a), and individual companies (Figure 3b), for all time points, are presented. Results show standing time significantly increased by 5% between baseline and one month follow-up for the total group (Z = −2.716, *p* = 0.007). In addition, a 4% increase in standing time between baseline and follow-up for Company 2 (Z = −2.207 *p* = 0.027). No other significant changes between baseline and follow-up were found for the total group or individual companies. 

**Figure 4:**
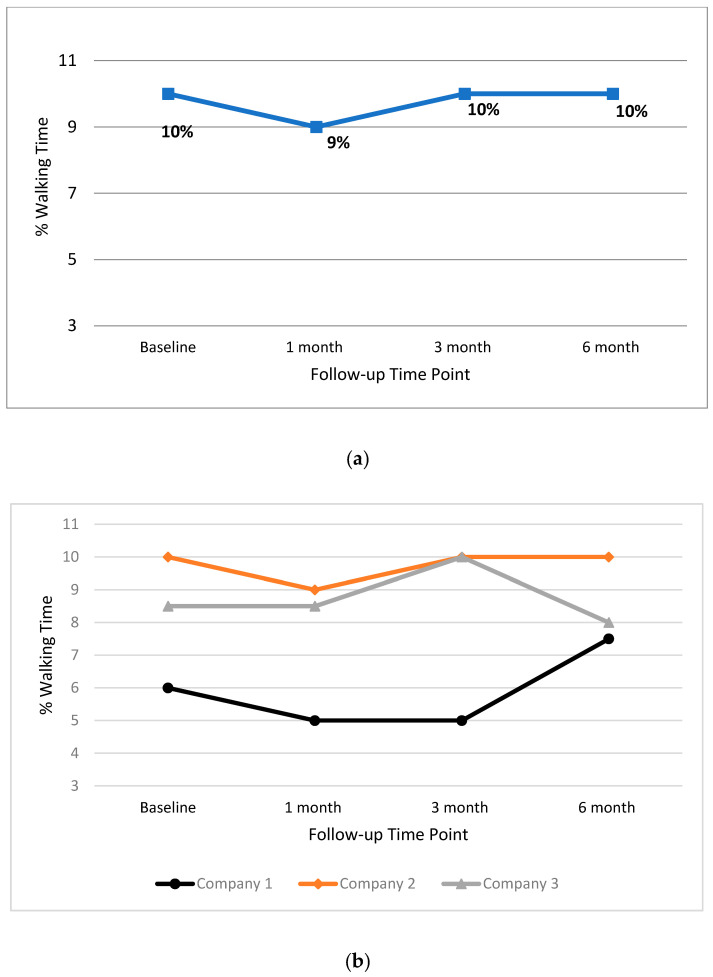
Median percentage workplace walking time for the total group (**a**) and individual companies (**b**) at baseline, one month, three month and six month time points.

In Figure 4, the median walking percentages for the total group (Figure 4a), and individual companies (Figure 4b), for all time points, are presented. Wilcoxon Sign Rank test results indicated that there were no significant changes in median scores for walking at any time point for the total group or for individual companies. 

**Figure 5:**
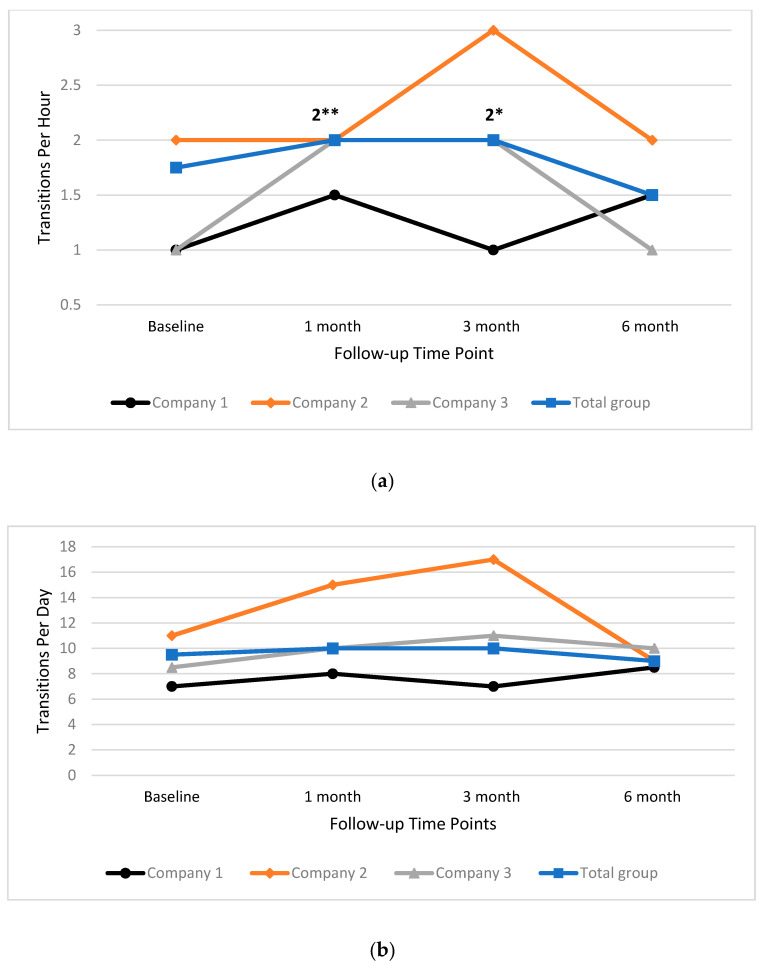
Median transitions per hour (**a**) and day (**b**) for total group and individual companies at baseline, one month, three month and six month time points. ** = significant change, *= trend towards significant change.

In Figure 5, the median values for transitions per hour (Figure 5a), and per day (Figure 5b), are presented for each time point. Results show that transitions per hour significantly increased in Company 3 by 1.00 between baseline and one month follow-up (Z = −2.554, *p* = 0.011). This increase remained stable and trended close to significant at three month follow-up (Z = −2.333, *p* = 0.02). No other significant changes between baseline and follow-up were found for the total group or individual companies. 

##### Musculoskeletal Pain

**Figure 6:**
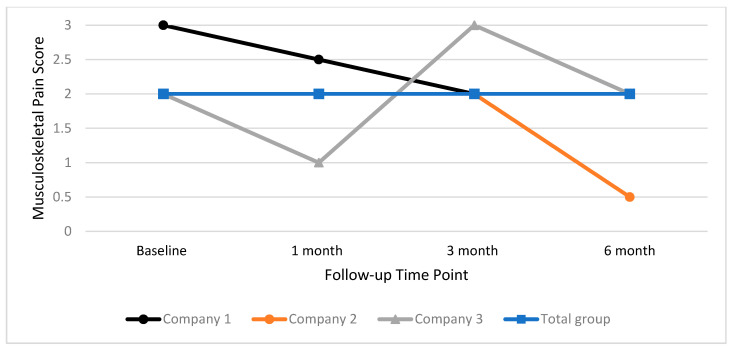
Median musculoskeletal pain score for total group and individual companies at baseline, one month, three month and six month time points.

Figure 6 illustrates the median scores for self-reported musculoskeletal pain. Results of the Wilcoxon Sign Rank test indicate that there were no significant changes in median pain scores at any time point for the total group, and for individual companies.

##### Work Engagement

**Figure 7:**
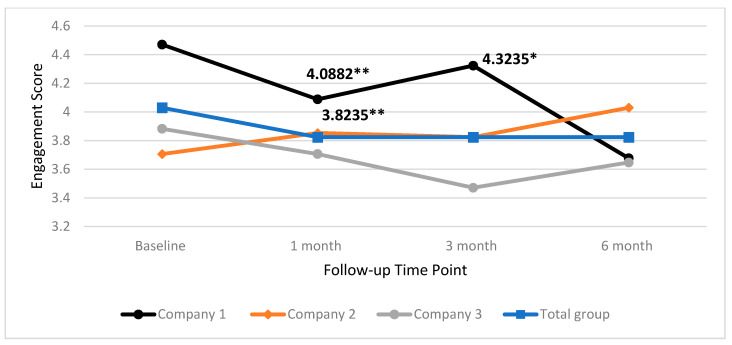
Median work engagement scores for total group and individual company at baseline one month, three month and six month time points. ** = significant change, *= trend towards significant change.

In Figure 7 the median scores for work engagement are presented, for both the total group, and individual companies. Results show that that work engagement significantly decreased by 0.2059 at one month follow-up for the total group (Z = −2.838, *p* = 0.005). Work engagement also decreased significantly in Company 1 by 0.3824 at one month follow-up (Z = −2.608, *p* = 0.009). This also trended towards a significant decrease at three month follow-up (Z = −2.197, *p* = 0.028). No other significant changes between baseline and follow-up were found for the total group or individual companies.

##### Health-Related Absenteeism

The mean hours missed for health for the total group and individual companies is presented in Table 9. The means are presented as all median values, and all but one IQR equalled zero. Results of the Wilcoxon Sign Rank test indicated that there was no significant change in health-related absenteeism for the total group or individual companies. 

#### 3.4.2. Additional Unintended Effects, and Facilitators and Barriers to Effectiveness

Several qualitative themes developed which highlighted participants’ and stakeholders’ perceptions of additional unintended effects of the intervention and facilitators and barriers of the effectiveness of the intervention. Table 10 shows that six themes developed which align to the effectiveness dimension. Example quotes are given to illustrate what participants and stakeholders shared in relation to each theme. 

### 3.5. Maintenance

All four companies maintained participation over six months. Each company self-reported information regarding institutionalisation of the digital intervention. Companies 2 and 4 have expressed interest in purchasing to institutionalise the intervention into existing health and wellbeing programming. Company 3 has purchased the digital intervention and expanded to all UK offices and Company 1 did not show interest in purchasing the digital intervention in its current form. 

Table 11 shows that two themes developed as a result of the analysis of the qualitative data relating to the RE-AIM dimension of maintenance. In the table the themes ‘Wellbeing important to company’ and ‘Need to create more buy-in with report on results at both individual and setting level’ are presented along with example quotes from participants and stakeholders which illustrate what was said in relation to each theme. 

## 4. Discussion

The aim of this study was to examine and understand the potential reach, effectiveness, adoption, implementation and maintenance of a digital health promotion application which targets reducing and breaking up sedentary behaviour across multiple workplace settings. 

The RE-AIM QuEST mixed methods framework facilitated a robust evaluation across 21 indicators of reach, effectiveness, adoption, implementation and maintenance. Upon analysis of this data it is evident that the digital application has the potential to be adopted by small to mid-sized companies and reach a large proportion of employees using minimal resources and company allocated time. The digital application positively affected sedentary behaviour by significantly increasing standing time and transitions per hour in Company 1 with no negative effects on musculoskeletal pain in the short term. Additionally, three out of four companies are willing to maintain and institutionalise the application into existing workplace wellbeing initiatives. However, with positive effects short lived, and several barriers identified across RE-AIM, significant improvements can be made to the Welbot digital application. Addressing the barriers which have been identified, while maintaining the positive attributes of the application will be critical to producing an effective digital application which also has the potential for scale-up across settings. The following sections will focus on what has been learned about the five RE-AIM dimensions, in a bid to understand how to improve each. Again, the RE-AIM dimensions order has been changed to reflect the chronological order of occurrence within the research process. 

### 4.1. Adoption

The companies that adopted the intervention were smaller, and required significantly less emails and meetings than larger companies who did not adopt the intervention. This suggests that there are barriers to larger companies implementing new health and wellbeing practices. Additionally, despite discussions of the high-level security features and compatibility of Welbot, larger companies that did not adopt the intervention reported concerns related to the IT security as a reason for not adopting the intervention. Evidence from other workplace health interventions indicates that to improve adoption, more assessments of the organisational culture may be needed to understand how to create “buy-in” at multiple levels of companies with complex management structures [16,38,39,40,41]. When recruiting larger companies, digital interventions may need to allocate further resources towards developing additional recruitment and engagement tactics aimed at building a relationship to create buy-in at all levels. If time is a limited resource, then targeting smaller to mid-size companies for recruitment may be warranted. 

A second reason reported for not adopting the intervention was that the company had existing health programming. Although digital interventions can be effective on their own [42], it may be important that they are also adaptable and flexible in design, so that they can be added easily into existing programming by individual companies. As they evolve and build in new content, digital interventions, like Welbot, may be uniquely adaptive to tailoring content to individual company needs and contexts [8]. Finally, in companies that adopted the intervention, employees reported that they believed their employers were concerned about employee health and wellbeing. At the moment little is known about how a company develops an appreciation of employee health. Research is needed to develop a deeper understanding of why some companies prioritise staff wellbeing, and others do not. Assessments of this are warranted as understanding this could be central to increasing adoption of health interventions in the workplace [41].

### 4.2. Reach

#### 4.2.1. Participation Rate

A large proportion (59%) of all employees signed up for the digital intervention. This is higher than other interventions in the workplace [39,43] and indicates that the sedentary behaviour (SB) intervention is considered accessible and feasible by employees. This is important to future scale-up, as companies may be more likely to engage with interventions that can be used by the majority of their workers. Uptake did differ substantially between companies with the smaller companies (Companies 1 and 4) having much higher recruitment than the other two companies. This supports the findings of a review of recruitment strategies which found that workplace studies with higher recruitment rates tended to target smaller cohorts of employees [43]. Additionally, Companies 1 and 4 also had management engagement compared to less management engagement in Companies 2 and 3. This aligns to other interventions in which the level of buy-in from management appeared to affect the participation rate within the intervention [16,39,44]. To improve future participation rates, it may be important in the future for digital interventions to build in strategies (e.g., targeted management incentives) to ensure management buy-in and participation.

#### 4.2.2. Dropout Rate

Without context, the dropout rate appears to be high, with only 31% of the study population still completing six month follow-up. Qualitative data revealed that Company 4 suffered a significant IT issue which meant that most of the participants failed to access the digital application following baseline data collection. In other research studies, people who did not download the product may have been eliminated as participants and therefore not included when calculating the dropout rate. However, in this study, it was important to be transparent and understand issues such as this, to aid improvement. This particular issue will be further examined in the implementation section of the discussion. Additionally, the dropout rate reported may be higher than perhaps really is the case as the rate was calculated based on the number of questionnaires completed at each time point, rather than the actual number of participants who continued to use the program. It is likely that some participants continued to use the application, but did not continue to complete the questionnaire. Collecting and analysing company and individual usage data could improve Welbot’s understanding of the dropout rate and engagement with the digital application.

#### 4.2.3. Facilitators and Barriers to Reach

One qualitative theme developed which suggested that participants in the intervention had an existing awareness that sitting was a health issue and this directly influenced their motivation to participate in the study. This adds to the existing evidence that awareness of sitting as a risk factor appears to be important to eliciting motivation [45,46,47]. To improve the reach (and adoption) of digital interventions like Welbot, more investment may be needed to create targeted educational content (e.g., short videos) which is clearly focused on both building knowledge about the associated health risks of sitting, and how reducing sitting time can improve health and wellbeing at work.

### 4.3. Implementation

#### 4.3.1. Cost

Indicators of cost within RE-AIM may be best explained as the financial investment and time needed to implement the intervention. In this evaluation, the estimated average financial cost per company was £175.50 and the estimated cost per participant was £8.78. This is significantly lower than the reported AU$431 or £230 per participant costs of a 12 month multi-component intervention, which installed standing desks costing AU$296 or £158 per participant. Furthermore, the estimated average company time used (4.5 h) to implement the intervention across four companies and 80 employees was presented. Reporting this time can give important insight into the labour costs which will be incurred by companies that adopt the intervention [48]. Given the relatively low estimated financial costs, and minimal hours used to implement the intervention Welbot may be considered “low cost”. Although knowing this information is important, it gives little insight into whether the companies themselves perceived this cost as affordable and acceptable. Two qualitative themes add this important contextual information and suggest that the companies perceived the implementation to be straightforward, and required minimal resources. This suggests that this digital intervention would require minimal resources to be widely implemented. This is in contrast to other types of workplace interventions that have been critiqued for being complex and expensive [14]. For example, Neuhaus et al.’s multi-component intervention was effective, however the authors acknowledged that participation was limited by funding and that findings may not be generalised across the wider population of workplace settings [49]. In here lies the balancing act of practical research. Do researchers continue to heavily resource interventions to produce an effect on behaviour which may not be generalisable or do researchers work within the constraints of the resources, to try to balance what is implemented, with the resources available in real-world office settings. We would argue that there is a need for a more balanced approach. An approach is needed that recognises that understanding the potential for real-world, wide-scale, implementation is important to understand and address this large-scale public health problem. 

#### 4.3.2. Facilitators and Barriers to Implementation

In addition to the two themes discussed above, in-house leadership within the companies appeared to be important to successful implementation. Participants and stakeholders both reported that a visible leader of the intervention helped to keep the implementation running smoothly. Interestingly, the intervention did not require or suggest leadership roles, yet they evolved naturally within three of the companies. This result is similar to other research studies in which team champions and visible leadership were reported as important to the success of the intervention [16,49].

The IT support became a significant aspect of implementation of the digital intervention. Three companies reported that their IT department supported the implementation, and this was straightforward. However, one company (Company 4) did not have sufficient IT support to overcome download issues and suffered significantly. In the end, only three participants overcame the barriers and downloaded the digital program. As mentioned above, this is reflected in the dropout rate. With regions of the world, and individual companies having varying degrees of data policy (e.g., European General Data Protection Regulation (GDPR)), and data security systems; it will be important for digital health interventions, such as Welbot, to align with policy, and build strategies to mitigate and overcome implementation barriers. 

### 4.4. Effectiveness

#### 4.4.1. Effects on Sedentary Behaviour

In relation to its effects on sedentary behaviour, the digital intervention increased standing time for the total group, and increased transitions per hour in Company 3 significantly in the short term. The digital intervention did not significantly reduce overall sitting time. As the Welbot application targeted breaking up sitting bouts with standing exercises and stretches, this can be almost expected and is similar to other prompt-based studies [50,51,52,53,54]. There is evidence that suggests that even these small changes in number of transitions per hour may be important in reducing the disease risk associated with uninterrupted bouts of sitting [55,56,57,58]. Individual company results revealed that Company 1 saw reverse effects; with an increase in sitting, and a decrease in standing. This result may be partly explained by the qualitative finding that Company 1 stakeholders did not allow employees to get up from their desks when they pleased. This finding is not unique to this intervention [44,46], and suggests that, despite talk of concern for employee health, managers may not always buy into health promotion as health promoters would expect. In this study, it may have acted as a significant barrier to office workers in Company 1 feeling free to engage with nudges. Recent research has suggested that it will be important that broader contexts of the office are understood, and organisational level barriers are addressed to improve the potential for sustainable change [16,59]

#### 4.4.2. Facilitators and Barriers to Effectiveness

Perceived lack of time was also a barrier for changing behaviour, suggesting that office workers do feel it is challenging to interrupt work-related tasks when busy. This is consistent across the qualitative literature [47], and both barriers suggest that it may take considerable shift in perception, for both employees and employers, to view health-related breaks as time well spent. 

Participants also felt that a limited variety or choice of nudges that specifically targeted sitting was a barrier to effectiveness on the primary outcomes. This finding is similar to Taylor and colleagues’ findings, in which office workers quickly tired of the provided health promotion break options, and called for more frequent change in the break routines provided, and more choice in the physical movement that was suggested [44]. To enhance effectiveness, similar nudge-based interventions may need to spend more time engaging in the development of material and find creative ways to expand the intervention content. For new digital interventions like Welbot, this may require a gradual approach in which new nudges are added as and when they are ready.

Additionally, participants shared that some exercises/stretches could be done while still sitting, and if not specially told to stand, they would often stay seated. Other researchers have noted the importance of being specific with instructions, learning that “taking breaks” often did not help office workers reduce sedentary behaviour, with participants more likely to choose a seated social or online break over an active break [60,61]. 

Both intervention participants and stakeholders suggested that more personal feedback on progress, both at the individual level and company level could have improved the experience of participation. More descriptive visual feedback, including data visualisation, may be very important for developing motivation and self-regulation for employees, and creating buy-in for companies [44,60,62,63]. 

#### 4.4.3. Effects on Secondary Outcomes

Musculoskeletal pain is associated with long term sick leave, risk of disability and disability retirement [64,65]. In this intervention there was no significant change in musculoskeletal pain or health-related absenteeism. This is in contrast to several studies in the workplace that reported reductions in musculoskeletal pain after reducing office-based siting time [66,67,68]. Both results may be partly explained by the relatively low mean age of participants, and the low baseline score for both musculoskeletal pain and health-related absenteeism. Future interventions in sedentary office workers should continue to measure both outcomes to widen understanding of the potential attenuating effect reducing sitting may have on musculoskeletal symptoms. In future larger studies, subgroup analysis of these secondary outcomes, based on the presence of additional risk factors of musculoskeletal pain (e.g., age or obesity), may be needed to understand effects.

#### 4.4.4. Additional Unintentional Effects

Employees and employers expressed that they believed the digital intervention helped to raise awareness in the company that sitting was a health issue they should be concerned about. Experts suggest that building awareness may be essential to building autonomous motivation, in which a person endorses or identifies with the value of performing a behaviour or health practice [63,69]. Office-based sedentary behaviour interventions may need to be more heavily focused on educational intervention components to help build sustained motivation. In this digital intervention, although some basic information is given, this may not be enough to build intrinsic value in the health behaviour [70]. In future iterations of this digital intervention, developing more in-depth educational prompts which target office workers’ understanding of the associated risks, and potential benefits of reducing sitting time may help elicit more sustained motivation for behaviour change [60,63]. 

The qualitative data also suggested that the intervention created a sense of social unity in the office. This aligns with results of several other interventions [16,47,71] aimed at reducing sitting time and should be considered an additional benefit to health promotion programs in the workplace, particularly in offices where group cohesion is vital to the delivery of business. Researchers and start-up companies, like Welbot, should seek to further explore how and why positive social outcomes develop so that they may be specifically targeted, and promoted to stakeholders, as additional benefits of health promotion programs. For example, in the future Welbot program, a group of nudges could specifically target improving social interaction whilst reducing sitting. This may help to create more buy-in for interventions aimed at reducing sitting time in the workplace. 

The intervention did appear to negatively affect worker engagement in the short term for the total group and Company 1. Research conducted on work flow has suggested that nudging workers at inopportune moments can negatively influence work engagement and productivity [72,73], and although there may have been other work-related factors affecting work engagement; addressing this issue is important to understand when best to prompt breaks. In a recent study, Luo and colleagues developed a prompting system which enabled workers to set up their preferred work and sitting break durations, to create healthy habits [60]. Their results indicated that, when compared to participants who did not set break times, participants who set consistent intended break duration had higher post study habit strength time [60], suggesting that the ability to create personalised routines could be important to the effectiveness of nudge-based interventions. Creating personalised options may also help to mediate negative effects on work engagement and productivity. Moving forward, nudge-based interventions, similar to Welbot, may need to balance the approach taken with engagement and productivity. This may be critical to sustaining long-term buy-in for sedentary behaviour interventions in workplaces.

### 4.5. Maintenance

Maintenance within RE-AIM is concerned with how behaviour change is maintained six months or more after the intervention, as well as long term sustainability of the intervention. No significant effects were maintained at six month follow-up. In the reach, implementation and effectiveness sections, barriers have been discussed in detail, and addressing these barriers will also be fundamental to maintaining participation and behaviour change. Additionally, as adaptations are introduced, continuing to measure across RE-AIM indicators will be important to track how adaptations to the Welbot application affect each dimension. Having employees complete questionnaires long-term may be burdensome, and as the digital application evolves, exploration of connectivity options to integrate participants’ existing movement data (e.g., wearable technology data) could be explored as a more accurate and sustainable data source to understand long term behaviour change [8]. 

In relation to sustainability of the intervention, all four of the companies used Welbot for the six month intervention, and all four were continuing to use Welbot up until Covid-19 working at home measures came into place in the United Kingdom (U.K.). Additionally, even though Company 4 had significant issues with installation, they, along with Company 2 are seeking to permanently adopt the Welbot program. This suggests that they feel confident they can overcome the IT-related issues which affected the implementation of Welbot during this intervention. Furthermore, Company 3 purchased Welbot to continue and expand use to all U.K.-based employees. 

#### Facilitators and Barriers to Maintenance

Participants from Companies 2, 3 and 4 perceived that employee health and wellbeing was important to their respective companies. This existing interest from company executives most likely helped to create the buy-in needed to adopt the program long-term. In contrast, as discussed in the effectiveness section, Company 1 managers were less receptive to employees engaging in the intervention, and the company has not expressed interest in engaging further. This suggests that interventions may also need to evaluate wider contexts of the office setting, such as assessing management support [41] and targeting company leaders to try and increase knowledge and understanding of the benefits to offering occupational health and wellbeing programs [16,39,40]. This may help to improve the potential for sustainability of behaviour change, and the potential institutionalisation of interventions [59]. 

Stakeholders in all four companies suggested that more feedback and data at the company level would be one way to improve buy-in for long-term use of the Welbot product. Producing a report on volume and frequency of use may help employers make decisions about maintaining and institutionalising digital health promotion applications like Welbot. 

### 4.6. Implications

Based on the discussion, recommendations for improving the digital application are presented in Table 12. Although they are specific to the Welbot application many of the RE-AIM recommendations could be used to improve other digital interventions which aim to implement their intervention at scale, across multiple settings. Using the RE-AIM evaluation framework to plan, implement and conduct the evaluation has enabled the research team to test for effectiveness simultaneously with testing the potential for impact in real-world settings. This style of dissemination is rarely seen; however, it has allowed the research team to report on effectiveness within the frame of the real-world resources. This comprehensive evaluation early in development should allow Welbot the ability to continue to understand and improve the digital application’s effectiveness without compromising the potential adoption, reach, implementation and maintenance of the intervention. This is in contrast to interventions which need to make substantial alterations to the intervention components to try and improve adoption, reach, implementation and maintenance, with no guarantee that the effects seen in early phases will match the newly adapted intervention effects [14,16,49,74]. 

## 5. Strengths and Limitations

There are several strengths to this study. Firstly, the implementation under real-world conditions has allowed the researchers to gain understanding of the intervention’s potential for wide scale implementation at an early stage of intervention development. This approach is novel, and this study may provide a template for other researchers seeking to understand the scale-up potential of sedentary behaviour interventions and other health-related interventions in the workplace. Secondly, using mixed methods to evaluate the intervention is a strength as it allowed the researchers to contextualise what happened, with how and why it happened. The ability to do this is critical to deciding what steps are needed to improve an intervention. Thirdly, using the RE-AIM Quest evaluation framework has been a strength as using the five dimensions, and the corresponding indicators of each dimension, helps to organise and pinpoint areas of an intervention which need improvement, allowing for the intervention value to be judged on all dimensions which are important to wide scale implementation/scale-up. The study has been limited by the small sample size, subjective measure of sedentary behaviour, lack of control group and dropout rate (details of which have been discussed in the paper). All of these played a part in limiting the statistical tests and interpretations of the effectiveness data. However, in relation to the objective measurement of SB and the lack of control group, as discussed above, the exclusion of these methods may have enhanced our understanding of the real world potential reach, implementation, adoption and maintenance of Welbot. Two employees suggested that the participant burden of the questionnaire was high, and although adjustments were made, limiting the data collection to just primary outcome data may be important to maximise data collection. 

## 6. Conclusions

The evaluation showed that the Welbot application has the potential to reach a large proportion of office workers with minimal office resources needed. Welbot should continue to improve the application using feedback to help further the potential for impact at the individual level and the setting level. Adapting the intervention and evaluating new components across RE-AIM may be important to improving the effectiveness and maintenance of behaviour change at the individual level, while preserving adoption, reach and maintenance at the setting level.

## Figures and Tables

**Table 1:** RE-AIM dimensions, indicators assessed and the data source used to measure or inform indicators.

Dimension	Indicator	Data Source/Measure
**Reach**	Participation rate (total and variation across sites)	Record and report # participating/# eligible
	Drop-out rate	Record and report # signed up/# completed assessment
	Reasons for non-participation	Interviews and focus groups
	Decline rate across office sites	Record and report
	Barriers/facilitators	Focus groups with participants Interviews with stakeholders
**Effectiveness**	Sedentary behaviour	Occupational Sitting and Physical Activity Questionnaire (OSPAQ) Two item breaks in sitting questionnaire
	Musculoskeletal pain	Nordic Musculoskeletal Questionnaire (NMQ)
	Productivity-engagement in work	Utrecht Work Engagement Scale (UWES)
	Absenteeism	Absenteeism and presenteeism questions of the World Health Organisation’s Health and Work Performance Questionnaire (HPQ)
	Additional unintended consequences; physical and/or psychological effects (positive or negative)	Focus groups with participants
	Barriers/facilitators of effectiveness. What are the conditions that lead to effectiveness or no effect? What adaptations are needed to improve effectiveness? (RE-AIM QuEST)	Focus group with participants Interviews with stakeholders
**Implementation**	Barriers/facilitators, contextual factors and processes underlying barriers/facilitators How do we address barriers?	Focus groups with participants Interviews with stakeholders
	Measure of cost (financial and time)	Individual company self-report and stakeholder interviews
**Adoption**	Rate of adoption	Record and report # approached, # declined and # enrolled
	What affects company participation/engagement	Interviews with stakeholders
	Method used to identify target deliver agent	Record and report
	Inclusion vs. exclusion criteria of delivery agents	Record and report
	Characteristics of setting and participants of adoption/non-adoption (drop-out participants/setting characteristics)	Record and report company characteristics
**Maintenance**	Outcome measurement six or more months from baseline (RE-AIM QuEST)	All questionnaires
	Is the program still in place and to what extent?	Record and report
	What are the barriers to maintaining the program?	Contact companies post-intervention reporting most up to date maintenance information possible Focus group participants Interviews with stakeholders

**Table 2:** Baseline descriptive characteristics of participants.

Characteristic	Valid Data	Mean (Standard Deviation)	Median (Interquartile Range)
Age (years)	80	33.8 (11.3)	29.5 (25.0, 40.3)
Height (cm)	79	169.8 (10.1)	169 (161,175)
Weight (kg)	76	71.6 (13.9)	70 (60.2,82.0)
BMI	76	24.8(4.2)	24.5 (22.4, 26.8)
Sitting (% workplace)	75	77.3 (14.9)	80 (70.0, 90.0)
Standing (% workplace)	75	10.8 (12.4)	5 (5.0, 10.0)
Walking (% workplace)	75	11.8 (7.5)	10.0 (5.0, 10.0)
Sit to stand transitions per hour	80	1.8 (1.1)	1.8 (1.0, 2.0)
Sit to stand transitions per day	80	11.5 (6.7)	10.0 (7.0, 15.0)
Hours missed for health (previous 4 weeks)	80	1.5(5.2)	0 (0, 1.0)
Total engagement	80	3.97 (0.68)	3.97 (3.53, 4.46)
Musculoskeletal pain	80	2.16 (2.00)	2.00 (0, 3.00)

**Table 3:** Qualitative themes relating to indicators of Adoption.

Adoption Themes	Facilitator or Barrier	Quotes
Company buy-in for wellbeing	Facilitator	Participant—“*We have a mental health pillar it’s driven by the people in the pillar who care…. like people do definitely care about it.*” Participant—“*It is quite high, wellness is quite high profile; we do have quite a lot of values, like mindfulness, we have done quite a lot of stuff on workplace wellbeing*.”

**Table 4:** Individual company participation rates.

Company	Eligible Office Based Employees Invited to Participate	Employees Who Signed Up	Participation Rate
Company 1	20	19	95%
Company 2	27	12	44%
Company 3	70	30	43%
Company 4	20	18	95%

**Table 5:** Individual company dropout rate.

Company	Total Employees Who Signed Up for Intervention	Total Employee Dropout Rate at One Month Follow-Up	Total Employee Dropout Rate at Three Month Follow-Up	Total Employee Dropout Rate at Six Month Follow-Up
Total group	80	40% (*n* = 32)	56% (*n* = 45)	68% (*n* = 54)
Company 1	19	37% (*n* = 7)	47% (*n* = 9)	57% (*n* = 11)
Company 2	12	25% (*n* = 3)	41% (*n* = 5)	50% (*n* = 6)
Company 3	30	20% (*n* = 6)	47% (*n* = 14)	67% (*n* = 20)
Company 4	18	89% (*n* = 16)	94% (*n* = 17)	94% (*n* = 17)

**Table 6:** Qualitative themes relating to indicators of Reach.

Reach Theme	Facilitator or Barrier	Quotes
Existing awareness that sitting is a health issue to address	Facilitator	Participant—“*I think that in an office job you’re always sitting down, and everyone knows that isn’t good for you to sit down all day.”*

**Table 7:** Estimated financial cost and time used to implement the intervention.

Company	Companies Estimated Financial Cost (£) (Six Months Use)	Company Time Used
Total	£702	18 h
Company 1	£171	1 h
Company 2	£72	4 h
Company 3	£270	10 h
Company 4	£189	3 h
Average	£175.50	4.5 h
Per-participant	£8.78	13.5 min

**Table 8:** Qualitative themes and relating to indicators of implementation.

Implementation Themes	Facilitator or Barrier	Quotes
Getting started was easy and straightforward	Facilitator	Participant—“*It was really easy, we just downloaded it.*” Participant—“*It wasn’t any time at all really*.”
Minimal company resources needed to improve	Facilitator	Stakeholder—“*It was pretty straight forward*” Stakeholder—“*Actually the impact on my time in setting this all up was fairly minimal*.”
In-house leadership helped	Facilitator	Stakeholder—“*We want to make this work so I felt like I was taking on the leadership aspect of that with Jenny certainly being like the advocate alongside that as well.”*
IT crucial to successful implementation	Barrier	Stakeholder—“*People originally had a lot of problems getting the software uploaded. To that point, I think we didn’t get nearly enough participants and they were even trying several times. So, I think that’s a definite hurdle*.” Stakeholder—“*Probably with the IT bit, that initial concern to how we actually got it into our systems*.”

**Table 9:** Mean health related absenteeism in hours for the total group and individual company at baseline, one month, three month and six month follow-up.

Title	Baseline	One Month	Three Month	Six Month
Total group	1.47 (SD = 5.2)	0.83 (SD = 2.957)	1.13 (SD = 1.93)	5.12 (SD = 12.54)
Company 1	0 (SD = 0)	0 (SD = 0)	0 (SD = 0)	0 (SD = 0)
Company 2	2.00 (SD= 4.75)	0 (SD = 0)	2.79 (SD = 4.78)	8.17 (SD = 11.21)
Company 3	1.07 (SD = 2.91)	1.63 (SD = 4.2)	1.20 (SD = 3.1)	8.78 (SD = 18.4)

**Table 10:** Qualitative themes related to indicators of effectiveness.

Effectiveness Themes	Additional Unintended Effects, Facilitator or Barrier	Quotes
Raised awareness and profile of workplace health	Additional effects (positive)	Participant—*“I think for me it made me more aware of how much I was sitting if that makes sense it made me want to stand up more but even if it wasn’t like prompting me to stand.”* Participant—“*There was a lot of helpful tips you know for stretches and things you wouldn’t necessarily think about doing so.”* Participant—*“It’s just awareness for me just how much I’ve been sitting but also just the stretches I’ve got a bad back just now so I’ve also had some exercises from the physio so it’s another wee reminder for me so yeah just more awareness.”*
Created social unity	Additional effects (positive)	Participant—“*A good thing is you know that other people are using it and you can kind of see other people in the office getting up and doing the exercises and there is a sense of we are all aware that like this is an…..that kind of communal shared thing is a definite benefit of it as well.”* Participant—*“We encourage each other to do things and maybe encourage and as you say seeing someone do it makes you think I better do it as well.”* Participant—*“What will happen is you will see someone else doing it, so you do it along with.”*
Limited variety and choice of nudges targeting sitting	Barrier	Participant—“*At the start you were a bit more active but as it goes on it gets a bit more repetitive.”* Participant—*“It always tells you to sit or stand it says that one you’re meant to stand up for but I’ve often found myself not standing up and just doing it sitting down.”* Participant—“*To even just change them up weekly just like a variety of stuff I think that will get me more involved.”*
Personal feedback on progress could have improved experience of participation	Barrier	Participant—“*For me if it was a bit more interactive I like to see like all stats if I could choose and see at the end of the day how much water I’d had and just like you know a bit more detail, you know?! You’ve done this many workouts throughout the week*.” Participant—“*I would like it to be able to track the feelings*.”
Perceived lack of time to engage with nudge	Barrier	Participant—*“You would be in the middle of something and you would be like okay I’ll pause it, not got time to do it because you are concentrating on something.”*
Company 1—rigid management style	Barrier	Stakeholder*—“Basically, like a call centre, yes. So, they’re not free to just get up and wander about, you know?”*

**Table 11:** Qualitative themes related to indicators of maintenance.

Maintenance Themes	Facilitator or Barrier	Quotes
Companies 2, 3 and 4—wellbeing important to company	Facilitator	Participant—*(P1): “Yeah, I think we are yes, I think the company are interested in the sort of how staff are how their wellbeing is.” (P2) replies: “Anything that kind of improves your wellbeing.”* Participant—“*It is quite a high…. wellness is quite high profile,….we do have quite a lot of values, sort of, like, mindfulness, we have done quite a lot of stuff on workplace wellbeing.*”
Need to create more buy-in with report on results at both individual level and setting level	Barrier	Stakeholder 1—“*For me the one thing that we haven’t seen that we would get with our employee assistance thing was for me to get as the gatekeeper, get some data on how much it’s being used*.” Stakeholder 2—“*The type of business we are it’s an analytical kind of company, so a lot of them like the detail and they’d like to almost see graphs in terms of movements and stuff*.”

**Table 12:** Recommendations to improve a digital application’s reach, effectiveness, adoption, implementation and maintenance.

RE-AIM Dimension	Recommendation
Adoption	Allocate resources towards developing additional recruitment and engagement tactics tailored for larger companies and district management levels aimed at building relationships and creating buy-in at all levels. Investigate and develop the adaptability of the application to add to existing workplace health programs and tailor to individual contexts. Investigate and assess how individual companies develop an appreciation of employee health.
Reach	Build in strategies (e.g., targeted management incentives) to ensure management buy-in and participation. Collect and analyse company and individual usage data to build understanding of the dropout rate and engagement with the digital application. Create targeted educational content (e.g., short videos) which is clearly focused on both building knowledge about the associated health risks of sitting, and how reducing sitting time can improve health and wellbeing at work.
Effectiveness	Allocate resources to the development and testing of creative ways to expand intervention content. This may require a gradual approach in which new nudges are added as and when they are ready. Specifically state in instructions the recommended posture and active nature of nudges as participants are more likely to choose a seated social or online break over an active break. Provide descriptive feedback, including data visualisation, to develop motivation and self-regulation within employees, and build buy-in with companies. Continue to measure both outcomes to widen understanding of the potential attenuating effect reducing sitting may have on musculoskeletal symptoms and absenteeism. In future larger studies, subgroup analysis of additional risk factors of musculoskeletal pain (e.g., age or obesity) may be warranted. Develop more in-depth educational nudges which target office workers’ understanding of the associated risks, and potential benefits of reducing sitting time which may help elicit more sustained motivation for behaviour change. Develop content to specifically target improving social interaction whilst reducing sitting. Build in further personalisation in relation to frequency and intensity of nudges.
Implementation	Allocate resources to building implementation strategies to mitigate potential barriers to implementation (e.g., I.T. implementation strategies). Promote and support companies in creating in-house leadership for the digital application. Produce estimates of financial cost and labour costs of the intervention.
Maintenance	Investigate potential to integrate existing movement data (e.g., data captured by wearable technology) as a data source to understand long-term behaviour change. Assess management support and target company leaders to try and increase knowledge and understanding of the benefits to offering occupational health and wellbeing programs. Use data to report on the volume and frequency of use of the digital application to employers.
